# Akute psychotische Störung als erste klinische Manifestation einer Multiplen Sklerose – eine Kasuistik

**DOI:** 10.1007/s00115-021-01107-y

**Published:** 2021-03-24

**Authors:** Sharmili Edwin Thanarajah, Hannah Wendy, Andreas Reif, Christine Reif-Leonhard

**Affiliations:** Klinik für Psychiatrie, Psychotherapie und Psychosomatik, Uniklinik Frankfurt, Heinrich-Hoffmann Str. 10, 60528 Frankfurt am Main, Deutschland

## Hintergrund

Multiple Sklerose (MS) ist die häufigste entzündliche Erkrankung des zentralen Nervensystems (ZNS) im jungen Erwachsenenalter. Weltweit sind mehr als 2,3 Mio. Menschen betroffen – Frauen doppelt so häufig wie Männer. Die Erkrankung ist gekennzeichnet durch eine autoimmunvermittelte Demyelinisierung im ZNS einhergehend mit motorischen, sensorischen und neuropsychiatrischen Defiziten.

Bereits Charcot beschrieb im 19. Jahrhundert auch psychiatrische Syndrome als Teil der Erkrankung. Am häufigsten treten Depressionen und Angststörungen auf [1]. Im Krankheitsverlauf können auch kognitive Defizite und organische Persönlichkeitsveränderungen hinzukommen [8]. Psychotische Symptome sind selten und spielen eher eine Rolle als Nebenwirkung der MS-Therapie mit Kortikosteroiden und seltener β‑Interferonen [5].

Bei unserer Patientin trat die akute psychotische Störung als erste klinische Manifestation der MS auf und führte zur Diagnosestellung.

## Kasuistik

Die 36-jährige Patientin wurde durch Polizei und Rettungsdienst vorgestellt. Der Ehemann berichtete, dass sich die Patientin seit ca. einer Woche zunehmend „merkwürdig“ verhalten habe. Sie habe sich sozial zurückgezogen, kaum gegessen, getrunken und geschlafen. Sie habe ihren Ehemann, von dem sie seit einem Jahr getrennt lebe, angerufen. Als er eintraf, habe sie ihn verkannt und sei schreiend zu den Nachbarn gelaufen.

Die Patientin war zunächst apathisch und beantwortete keine Fragen. Nach Einnahme von 1 mg Lorazepam berichtete die Patientin sich an die vergangene Woche nicht erinnern zu können. Ihr Ehemann habe sie vor 20 Jahren ins Koma legen lassen. Sie sei seit 20 Jahren schwanger. Der Vater des Kindes habe Suizid begangen und befinde sich in einer anderen Welt. Sie selbst habe ihr „altes Ich“ hinter sich gelassen habe und sei nun Gott.

Im Kontakt war die Patientin angespannt und distanzgemindert. Formalgedanklich war sie sprunghaft, phasenweise vorbeiredend. Es bestanden Wahngedanken in Form von Größen‑, Beziehungs- und Beeinträchtigungsideen mit hoher Wahndynamik. Ich-Störungen wurden verneint. Die Stimmung war wechselnd gehoben bis ängstlich. Der Antrieb war unauffällig. Die Psychomotorik war unruhig, angespannt. Das Krankheitsgefühl und die Krankheitseinsicht waren aufgehoben. Von akuter Suizidalität war die Patientin bei Aufnahme klar distanziert.

Die Patientin wurde zunächst freiwillig auf die geschützte Station aufgenommen. Bei fehlender Krankheitseinsicht und Behandlungsmotivation wurde eine Eilbetreuung eingerichtet und die Patientin nach § 1906 BGB untergebracht.

Die Patientin ließ sich im Verlauf auf die antipsychotische Behandlung mit Risperidon ein; die Dosis wurde stufenweise auf 4 mg/Tag gesteigert. Hierunter zeigte sich eine rasche Regredienz der Symptomatik. Die Patientin war zunehmend kooperativ und krankheitseinsichtig.

In der Reevaluation fand sich kein Hinweis für neurologische oder psychiatrische Symptome in der Vergangenheit. Vorerkrankungen und Vormedikation wurden verneint. Die Suchtmittel- und Familienanamnese blieben unauffällig. In der neurologischen Untersuchung fand sich kein pathologischer Befund. Die Labordiagnostik war unauffällig. Die von der Patientin geäußerte wahnhafte Vermutung einer Schwangerschaft konnte bei fehlender β‑HCG-Wert-Erhöhung ausgeschlossen werden.

**Figure 1:**
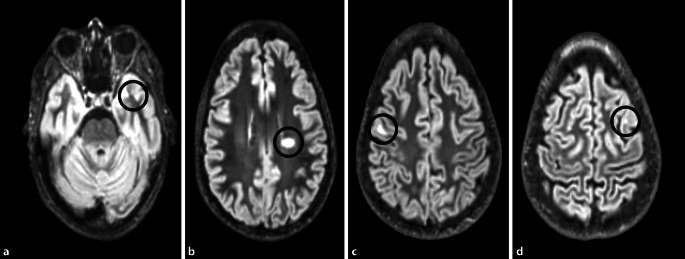

In der kraniellen Magnetresonanztomographie (cMRT; Abb. 1) wurden bei Auffälligkeiten in der standardmäßig erhobenen fluid attenuated inversion recovery(FLAIR-)Sequenz sowie der Kontrastmittel(KM)-Sequenz (T1) noch eine Double-inversion-recovery(DIR)-Sequenz sowie eine diffusionsgewichtete Sequenz ergänzt. Hier zeigten sich insgesamt supra- und infratentoriell multiple periventrikuläre und juxtakortikale Läsionen; darunter war eine periventrikuläre Läsion schrankengestört. Im MRT des Myelons zeigte sich eine Läsion links paramedian auf Höhe BWK 8. Die visuell evoziierten Potenziale waren beidseits leichtgradig latenzverzögert. Die motorisch und somatosensibel evozierten Potenziale waren unauffällig. Die Liquordiagostik zeigte keine Pleozytose. Es ließen sich oligoklonalen Banden vom Typ III nachweisen. Die antineuronalen Antikörper (Anti-Aqp 4, Glutamatrezeptoren [Typ NMDA], CASPR2, Anti-Hu, Ri, ANNA2, Yo, Tr/DNER, Myelin, Ma/Ta, GAD65, Amphiphysin, Glutamatrezeptoren [Typ AMPA], GABA-b-Rezeptoren, LGI1, ZIC4, DPPX, CARPVIII, Glycinrezeptoren, mGluR1, mGluR5, GABA-a-Rezeptoren, „Rho GTPase activating protein 26“, ITPR1, Homer 3, MOG, Recoverin, Neurochondrin, GluRD2, Flotilin-1/2, IgLON5, Neurexin-3‑α, ERC1, Sez612, AP3B2, Contactin1, Neurofascin 155, Neurofascin 186, AT1A3, KCNA2, Dopaminrezeptoren) waren negativ. Die Erregerdiagnostik (Lues, Borrelien, HCV, HBV, HAV, HIV, CMV, HSV, EBV, VZV) blieb ohne pathologischen Befund. In der Labordiagnostik fiel eine Erhöhung von DNS-AK auf. Der Bestätigungstest war negativ und die ANA-Titer waren nur leicht erhöht, sodass wir von einer reaktiven Antikörpererhöhung ausgingen. Es bestand klinisch und anamnestisch kein Hinweis für einen systemische Lupus erythematodes.

In Zusammenschau der Befunde diagnostizierten wir eine MS. Die akute psychotische Störung werteten wir als ersten klinisch-manifesten Schub. Wir führten über drei Tage eine Hochdosis-Kortionsontherapie durch. Diese wurde von der Patientin gut vertragen und sie war anschließend beschwerdefrei.

Wir klärten die Patientin über eine Basistherapie mit Glatirameracetat, Teriflunomid oder Dimethylfumarat auf. Aufgrund der psychotischen Episode waren wir hinsichtlich β‑Interferonen zurückhaltend. Wir empfahlen die Fortführung der Behandlung mit Risperidon für mindestens sechs Monate.

## Diskussion

Patienten mit MS entwickeln im Krankheitsverlauf in über 50 % neuropsychiatrische Auffälligkeiten [6]. Depressive Symptome sind in der Mehrzahl, Psychosen treten in populationsbasierten Studien mit 2–3 % selten auf. In 90 % der Fälle geht die MS-Diagnose der Psychose voraus. Dieser Fall zeigt auf, wie wichtig die diagnostische Abklärung jeder erstmalig aufgetretenen psychotischen Symptomatik ist, um eine hirnorganische Genese adäquat zu behandeln. Unsere Patientin zeigte einen akuten Beginn und eine rasche Progredienz der polymorph psychotischen Symptomatik bis hin zum Vollbild einer akuten psychotischen Störung mit schwerer formaler Denkstörung, Wahn und starken affektiven Schwankungen. Allerdings fanden sich keine sonstigen neurologischen Defizite. Erst durch cMRT und Liquordiagnostik konnte die Diagnose gesichert und eine immunsupressive Therapie eingeleitet werden, worunter sich die Symptomatik regredient zeigte.

Es ist unklar, über welche Mechanismen die entzündlichen Läsionen der weißen Substanz die psychotische Symptomatik bedingen. Einige Fallberichte diskutierten, dass eine kritische Veränderung der funktionellen Konnektivität durch primär frontotemporal und periventrikulär verteilte Läsionen der psychotischen Symptomatik zugrunde liegt [4]. Beim Auftreten psychotischer Symptome im Laufe der Erkrankung sollte der gesteigerte Cannabismissbrauch unter MS-Patienten berücksichtigt werden, da Tetrahydrocannabinol bei Prädisposition Psychosen auslösen oder deren Auftreten beschleunigen kann [6].

Die Drogenanamnese unserer Patientin war blande. In unserer Kasuistik fand sich eine frontotemporale Betonung der Läsionen. Die KM-aufnehmende, frische Läsion war periventrikulär lokalisiert. Auch wenn die zeitliche Assoziation zwischen Symptombeginn und der frischen Läsion einen ursächlichen Zusammenhang wahrscheinlich macht, ist es möglich, dass hier zwei unabhängige Erkrankungen vorliegen und der MR-Befund einem Zufallsbefund entspricht.

Für einen ursächlichen Zusammenhang spricht auch das gute Ansprechen auf die antipsychotische Medikation und die Kortisontherapie. Unter der Steroidbehandlung kam es nicht zu einer Exazerbation der psychotischen Symptomatik – dies deckt sich mit bisherigen Fallberichteten [2]. Es gibt keine Metaanalyse zur Wirksamkeit von Antipsychotika bei MS-Patienten. Aufgrund des besseren Nebenwirkungsprofils werden Antipsychotika der zweiten Generation empfohlen [3]. Für Risperidon und Quetiapin wurden krankheitsmodifizierende Effekte im Tiermodell gezeigt [7, 9].

Zusammenfassend illustriert unsere Kasuistik die Notwendigkeit der vollständigen Diagnostik inklusive cMRT bei jeder ersten psychotischen Episode auch ohne sonstiges fokal-neurologisches Defizit. Die adäquate Therapie neuropsychiatrischer Symptome ist entscheidend für den Krankheitsverlauf und die Lebensqualität der MS-Patienten.
